# High neuropilin and tolloid‐like 1 expression associated with metastasis and poor survival in epithelial ovarian cancer via regulation of actin cytoskeleton

**DOI:** 10.1111/jcmm.15547

**Published:** 2020-07-08

**Authors:** Yunzhao Xu, Wei Wang, Jinling Chen, Haixia Mao, Yuanlin Liu, Shuting Gu, Qinqin Liu, Qinghua Xi, Wenyu Shi

**Affiliations:** ^1^ Department of Clinical Biobank Affiliated Hospital of Nantong University Nantong China; ^2^ Department of Pathogen Biology School of Medicine Nantong University Nantong China; ^3^ Department of Obstetrics and Gynecology Affiliated Hospital of Nantong University Nantong China; ^4^ Department of Oncology Affiliated Hospital of Nantong University Nantong China

**Keywords:** actin cytoskeleton, bioinformation, epithelial ovarian cancer, KIF2A, NETO1

## Abstract

Abnormal expression of neuropilin and tolloid‐like 1 (NETO1) has been detected in some human carcinomas. However, the expression of NETO1 and the underlying mechanism in epithelial ovarian cancer (EOC) remain unknown. In this study, we found that a higher NETO1 expression in EOC tissue samples compared to normal ovarian tissue samples was significantly correlated with worse overall survival. Additionally, Cox regression analysis suggested that NETO 1 was independently associated with overall survival. NETO1 overexpression enhanced the EOC cells’ migration and invasion capability in vitro via regulation of actin cytoskeleton. Mechanistically, silencing NETO1 reduced the expression of β‐tubulin, F‐actin and KIF2A. In conclusion, our results demonstrated the critical role of NETO1 in EOC invasion, and therapies aimed at inhibiting its expression or activity might significantly control EOC growth, invasion and metastatic dissemination.

## 1 INTRODUCTION

Although patients who are diagnosed with epithelial ovarian cancer (EOC) immediately receive necessary surgery and then post‐operative six cycles of periodic chemotherapy, the overall survival rate is still below 50%.^1^ Some EOC patients are detected at the stage of extensive metastasis because they may have no symptoms or atypical manifestations.^2^ Therefore, there is insufficient understanding of the early events in the development of EOC as well as lack of sensitive techniques for detection in the early stage leading to inadequate clinical treatment. However, even with similar clinical manifestations and the same stage, different patients have variable clinical outcomes.^3^ Owing to the molecular heterogeneity,^4^ a considerable number of EOC patients may experience earlier metastases and relapse compared to other patients. Accordingly, in the age of ‘big‐data’ and ‘precision medicine’,^5^ there is a critical need for highly sensitive prognostic biomarkers for EOC patients.

NETO1 (neuropilin and tolloid‐like 1), a protein coding gene, encodes a transmembrane protein containing two extracellular CUB domains. Research has revealed that this gene serves as an ionotropic glutamate receptor and can encode a protein that is involved in spatial learning and memory by regulating the function of neuronal N‐methyl‐D‐aspartate receptor complexes in the hippocampus. An important paralog of NETO1 is NETO2. Recent studies have shown that NETO2, which is primarily implicated in neuron‐related processes, is expressed in diverse human tumour types. A five‐gene hepatic signature including NETO2 may play a feasible role in the prediction of tumour growth and risk of death in patients with hepatocellular carcinoma (HCC), suggesting a useful molecular tool for therapeutic management of HCC patients.^6^ A recent study identified 21 genes including NETO1 that could promote bowel metastases in EOC and evaluated their potential significance as molecular targets for treatment of advanced EOC patients.^7^ Taken together, these findings imply that NETO1 might contribute to carcinogenesis, tumour progression and poor clinical outcomes. However, NETO1 expression in EOC patients and its relationship with clinicopathological features have not been evaluated till date. Moreover, mechanisms involving NETO1 that underly EOC progression remain to be elucidated.

Therefore, in this study, both traditional laboratory studies and bioinformatic analysis were performed to investigate the potential role of NETO1 in carcinogenesis, invasion and progression in EOC patients. Furthermore, we chose the regulation of actin cytoskeleton^8^, ^9^, ^10^ as the possible signalling pathway that might be involved in the development of EOC via bioinformatics analysis. A potential molecular target may help in selecting therapeutic markers in EOC patients with malignant bowel obstruction as well as prolong the survival time and improve the quality of life of these patients.

## 2 MATERIALS AND METHODS

### 2.1 Patient tissue samples

All specimens were collected by specialized personnel from patient visited Gynecology Department and given informed consent, Affiliated Hospital of Nantong University between July 2005 and July 2010. A total of 13 cases of normal ovarian tissues, resected from double adnexal hysterectomy, and 98 cases of ovarian cancer tissues were gathered and detected in our study. There were 69 cases of serous carcinoma and 29 cases of other types for tumour classification, and 60 cases of stage I and 38 cases of stage II‐IV regarding of FIGO stage in total 98 cases. Detailed clinicopathological materials including patients’ age, tumour grade, 5‐year survival outcome follow‐up and some other information were shown in Table 1. The protocol of selecting patients’ samples in our study was described as before. All patients were followed up from the time of being diagnosed till July 2019. The protocol of obtaining clinical samples was approved by the Ethics Committee of Affiliated Hospital of Nantong University.

**TABLE 1:** NETO1 expression in normal ovarian and EOC tissue samples

Samples	n	NETO1 expression			
		Low or none	High	Pearson *χ* ^2^	*P*‐value
Normal ovarian	20	20 (100%)	0 (0%)	6.1485	0.013*
EOC	98	74 (75.51%)	24 (24.49%)		

Abbreviations: EOC, epithelial ovarian cancer; NETO1, neuropilin and tolloid‐like 1.

* *P *< 0.05.

### 2.2 TMA construction and IHC analysis

The NETO1 protein expression was detected by using the tissue microarray (TMA) system to perform immunohistochemistry (IHC) analysis in 98 cases of EOC tissues. The method of TMA construction has been previously described. Immunostaining was performed using rabbit monoclonal antibody to NETO1 (1:200; Abcam, Cambridge, MA, USA) as the primary antibody and then incubated with the secondary antibody (SantaCruz, CA, USA). Phosphate buffered saline severed as negative controls (NCs). Two independent pathologists evaluated and scored the NETO1 semiquantitative expression of each slide without knowing the clinical information of the case. We took the staining intensity and the percentage of positively staining cells into account for the scoring method, which demonstrated previously. The final immunostaining score was evaluated by the following criteria: staining intensity scores (no staining = 0, weak = 1, medium = 2, strong = 3) and positive staining ratio of NETO1 (ranged between 0 and 100). The total score (staining intensity score × positive staining ratio) of NETO1 expression was divided into low expression and high expression groups. The X‐tile software program was performed to set the cut‐off point (99) for the NETO1 expression scores which was of statistically significance in terms of survival outcome. The expression scores were as below: low expression 0‐99 and high expression 100‐300 for NETO‐1 in EOC tissues.

### 2.3 Cell lines and culture conditions

In our study, five cell lines were used as follows: normal ovarian surface epithelium cell line IOSE80, EOC cell lines HO‐8910, A2780, SK‐OV3 and OVCAR‐3. A2780, OVCAR‐3 and SK‐OV3 were cultured in DMEM containing 10% foetal bovine serum (FBS). HO‐8910 and IOSE80 were grown in RPMI 1640 with 10% foetal bovine serum. The five cell lines were cultured in a 5% moisture CO_2_ and 37°C condition.

### 2.4 Western blot analysis

First, we obtained total protein from each cell line and measured the protein concentration using the BCA Protein Assay Kit with bovine serum albumin as the standard (Thermo Fisher Scientific, Waltham, MA, USA). Then, equal amounts of protein were separated and transferred to PVDF membranes at 300 mA for 2 hours. After blocking, we incubated PVDF membranes with primary antibodies overnight at 4℃: anti‐NETO1 antibody (1 μg/mL, ab111374; Abcam) and anti‐KIF2A antibody (1 μg/mL, ab55383; Abcam). A secondary horseradish peroxidase‐conjugated goat antimouse immunoglobulin G (IgG; 1:5000, ZB2305, Cambridge) or anti‐rabbit IgG (1:5000, ZB2301, Cambridge) was used for the membranes to incubate with at 37°C for 1 hour.

### 2.5 Interfering with NETO1

Four different shRNA sequences targeting NETO1 (shRNA 1‐4) together with NC (shRNA‐NC) were designed by Invitrogen online software BLOCK‐iTTM RNAi Designer. ShRNA target sequences were as follows:

shRNA‐1, sense: 5′‐ATGCAGAGGGAGGTATCTTTA‐3′;

shRNA‐2, sense: 5′‐CCGTCTTGGGAGTGCAAATTT‐3′;

shRNA‐3, sense: 5′‐CTGATATGTTCATTTCGTAAT‐3′;

shRNA‐4, sense: 5′‐CGATACAATTTCACACCTGAT‐3′;

shRNA‐NC, sense: 5′‐TTCTCCGAACGTGTCACGT‐3′.

According to the manufacturer's instructions, cells were transfected with Lipofectamine^™^ 3000 (3 μL/well; Invitrogen, Carlsbad, California, USA) and then added to the six‐well plate for 4 hours After cultured in fresh complete medium for 24 hours, the interference efficiency of NETO1 gene was detected by Western blot.

### 2.6 Invasion and migration assays

Transwell Boyden chambers (Corning, NY, USA) were utilized to analyse cell invasion and migration assays. Cells in each group (1 × 10^5^) were added to the top of the chamber with RPMI‐1640 alone, while the bottom chamber was filled with RPMI‐1640 containing 10% FBS. After 24‐hours incubation in a 5% CO_2_ and 37°C environment, the upper chambers were washed; the filter membranes were fixed using 4% paraformaldehyde for 30 minutes at 4°C and stained using 0.1% crystal violet for 5 minutes at 37°C.

We used wound healing migration assay to evaluate migration ability. Cells were harvested in the logarithmic growth phase, adjusting cell concentration to 2.5 × 10^5^ cells/mL, then seeded in a 24‐well plate with 1 mL per well, and cultured at 37°C in a 5% CO_2_ incubator. Cells transfected with shRNA‐NC and shRNA‐1, respectively, were cultured for 48 hours, and then, we used a 200 μL pipette tip to prepare a mechanical damage model. The damaged cell debris was washed gently by PBS, and after adding the fresh medium, the cells’ reaction was observed under an inverted phase contrast microscope.

### 2.7 GSEA enrichment analysis

Gene set enrichment analysis (GSEA), a calculation method that could estimate whether a list of previous defined genes shows concordant differences with statistically significant between two biological processes.^11^ This study employed the GSEA to illuminate the significant difference in survival rates observed between the low and high NETO1 groups after initially generating a sequential list of all genes according to their relationship among NETO1 expressions. For each analysis, the gene set permutations were performed 1000 times. The phenotype label was identified in the level of the NETO1 expression. In order to sort out the pathways enriched in each phenotype, the Normalized Enrichment Score (NES) and the nominal *P*‐value were utilized.

### 2.8 Immunofluorescence staining

Cells were seeded in a 24‐well plate, incubated overnight and fixed with 4% paraformaldehyde for 15 minutes. Cells were permeabilized with 0.1% Triton at room temperature for 15 minutes and then blocked with 5% FBS for 15 minutes. Next, cells were incubated at 4°C overnight with anti‐NETO1 (1 μg/10^6^ cells) or anti‐KIF2A (2.5 μg/10^6^ cells), anti‐β‐tubulin (1:50) and anti‐F‐actin (1:1000). After PBS washing, the cells were then incubated with goat anti‐rabbit FITC (1:200, SA00003‐2; Proteintech, Chicago, Illinois, USA) or goat antimouse CY3 (1:200, SA00009‐1; Proteintech, Chicago, Illinois, USA) for 1 hour at room temperature. Finally, nuclei were labelled with Hoechst dye for 15 minutes. Images were acquired by fluorescent microscope.

### 2.9 Statistical and multivariate analysis

SPSS20 statistic software (SPSSInc, Chicago, IL, USA), STATA 12.0 (Stata Corp, College Station, TX, USA) and X‐tile software were utilized in our study. Chi‐squared tests were performed to evaluate relationships between NETO1 expression and clinic‐pathologic characteristics. Clinicopathological parameters associated with patients’ outcome survival using Cox regression. Multivariate Cox regression model was adopted to evaluate the prognostic significance of NETO1 expression along with other clinical characteristics. Statistical calculations of NETO1 expression were performed using *t* test. A *P*‐value < 0.05 was recommend of statistical significance concerning the above analyses.

## 3 RESULTS

### 3.1 Clinicopathological parameters of EOC

**FIGURE 1:**
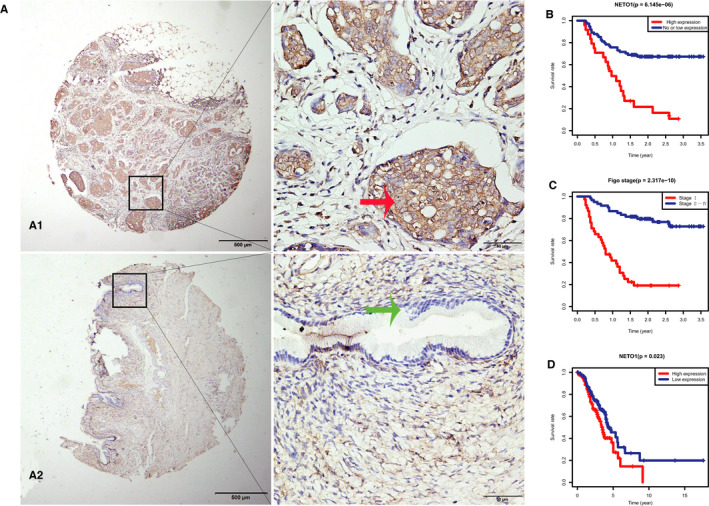
Expression of neuropilin and tolloid‐like 1 (NETO1) in epithelial ovarian cancer (EOC) and its relationship with prognosis. A, Positive immunohistochemical (IHC) staining for NETO1 was observed in tumours samples from EOC patients (A1), whereas IHC staining was negative in normal ovarian tissue (A2). Original magnification is × 40 in left images of A1, A2 (scale bars 500 μm); ×400 in right images of A1, A2 (scale bars 50 μm). B, Kaplan‐Meier plots using the log rank survival test were analysed. Survival curve based on our data shows that EOC patients with high NETO1 expression have a worse prognosis than patients with low and no NETO1 expression (*P* < 0.01). C, Survival curve based on our data demonstrates that EOC patients with high FIGO stage have a worse prognosis than patients with low FIGO stage (*P* < 0.01). D. Survival curve based on GSE9891 downloaded from the GEO database shows that EOC patients with high NETO1 expression have a worse prognosis than those with low NETO1 expression (*P* < 0.01)

Immunohistochemical staining was performed to detect NETO1 expression in EOC tissue specimens. NETO1 overexpression was observed in 24 (24.49%) cases of EOC but was absent in normal ovarian tissue specimens (*χ* ^2^ = 6.1485, *P* = 0.013; Table 1; Figure 1A).

Subsequently, we analysed the relationship between NETO1 expression and clinicopathological variables of EOC to find out the potential effects of NETO1. It was discovered that NETO1 protein expression level was significantly associated with FIGO stage (*χ* ^2^ = 7.536, *P* = 0.006), positive ascitic cells (*χ* ^2^ = 8.498, *P* = 0.004), metastasis (*χ* ^2^ = 9.932, *P* = 0.002) and serum CA125 level (*χ* ^2^ = 4.887, *P* = 0.027; Table 2).

**TABLE 2:** Neuropilin and tolloid‐like 1 (NETO1) expression associated with clinicopathological characteristics

Groups	n (98)	NETO1 expression			
		Low or none (74)	High (24)	Pearson *χ* ^2^	*P*‐value
Age at diagnosis				3.555	0.059
≤60 y	57	47	10		
>60 y	41	27	14		
FIGO stage				7.536	0.006*
1	60	51	9		
2‐4	38	23	15		
Tumour grade				0.028	0.867
Low	27	20	7		
High	71	54	17		
Tumour type				1.415	0.493
Serous	69	50	19		
Other types	29	24	5		
Ascites				0.579	0.447
No	47	37	10		
Yes	35	25	10		
Unknown	16	12	4		
Ascites cell				8.498	0.004*
No	54	45	9		
Yes	20	10	10		
Unknown	24	19	5		
Lymphatic metastasis				0.104	0.747
No	84	65	19		
Yes	14	9	5		
Metastasis				9.932	0.002*
No	63	54	9		
Yes	35	20	15		
Serum CA‐125 (U/mL)				4.887	0.027*
≤100	11	11	0		
>100	62	42	20		
Unknown	25	21	4		

* *P* < 0.05.

### 3.2 Survival outcome and multivariate analysis

The overexpression of NETO1 was associated with advanced cancer biology, indicated by metastasis, FIGO stage and serum CA125 level. Kaplan‐Meier curve analysis showed that EOC patients with a higher NETO1 expression and advanced stage had a poor overall survival (log rank, *P* < 0.001, Figure 1B,C).

On univariate survival analysis, NETO1 overexpression (HR 3.643; *P* < 0.001), older age (HR 2.293; *P* = 0.007), FIGO stage (HR 6.347; *P* < 0.001), tumour grade (HR 3.194; *P* = 0.008) and metastasis (HR 7.105; *P* < 0.001) were found to be associated with overall survival (Table 3). On multivariable survival analysis, NETO1 overexpression (HR 2.089; *P* = 0.027) and FIGO stage (HR 4.060; *P* < 0.001) were identified as independent predictive factors for poor clinical outcome (Table 3).

**TABLE 3:** Univariate and multivariate Cox proportional hazard models of overall survival

Variable	Univariate analysis			Multivariate analysis		
	HR	*P‐*value	95% CI	HR	*P‐*value	95% CI
NETO1						
Low vs high	3.643	0.000*	1.997‐6.645	2.089	0.027*	1.089‐4.005
Age (y)						
<60 vs ≥ 60	2.293	0.007*	1.260‐4.173	1.096	0.781	0.574‐2.096
FIGO stage						
I vs II‐IV	6.347	0.000*	3.317‐12.146	4.060	0.000*	1.883‐8.754
Grade						
Low vs high	3.194	0.008*	1.345‐7.586	1.657	0.300	0.637‐4.310
Tumour type						
Serous vs others	0.759	0.393	0.402‐1.430			
Ascites						
Yes vs no	1.340	0.356	0.720‐2.493			
Ascites cell						
Yes vs no	1.466	0.260	0.753‐2.855			
Lymphatic metastasis						
Yes vs no	1.989	0.080	0.922‐4.293			
Metastasis						
Yes vs no	7.105	0.000*	3.723‐13.562			
Serum CA‐125 (U/mL)						
<100 vs ≥ 100	2.187	0.196	0.668‐7.156			

Abbreviations: CI, confidence interval; HR, hazard ratio; NETO1, neuropilin and tolloid‐like 1.

* *P* < 0.05.

To confirm that NETO1 overexpression may serve as an independent predictive factors for poor outcome in EOC patients, bioinformatics analysis was performed. As shown in Figure 1D, survival curve based on GSE9891 ^12^ downloaded from the GEO database shows that EOC patients with high NETO1 expression have a worse prognosis than those with low NETO1 expression (*P* = 0.0023). Additionally, univariate analysis revealed that high NETO1 expression correlated significantly with a worse overall survival (HR 2.773; *P* = 0.001). The prognosis of ovarian serous carcinoma was worse than other subtypes. Other clinicopathological parameters associated with poor outcome included advanced stage, older age, platin treatment (Table 4a). In case of multivariate analysis, NETO1 expression remained independently associated with overall survival (HR 2.499, *P* = 0.009) along with the disease stage (Table 4b).

**TABLE 4:** A, Correlation between clinicopathological characteristics and overall survival in GEO patients using Cox regression. B, Multivariate survival model after variable selection

Clinicopathological variable	HR (95% Cl)	*P*‐value
A		
Histological subtype (serous vs others)	8.691 (1.212‐62.322)	0.031
Stage	2.152 (1.490‐3.109)	0.000
Grade	1.337 (0.975‐1.835)	0.072
Patient age (y)	1.026 (1.006‐1.047)	0.010
Platin treatment (yes vs no)	0.252 (0.080‐0.795)	0.019
Taxol treatment (yes vs no)	1.323 (0.886‐1.976)	0.171
Neoadjuvant treatment (yes vs no)	0.665 (0.320‐1.368)	0.268
NETO1	2.773 (1.509‐5.093)	0.001
B		
Stage	1.810 (1.226‐2.670)	0.003
Patient age (y)	1.026 (1.005‐1.047)	0.014
NETO1	2.499 (1.256‐4.973)	0.009

Abbreviation: HR, hazard ratio; NETO1, neuropilin and tolloid‐like 1.

### 3.3 Metastasis‐related cancer biology including cell migration and invasion

**FIGURE 2:**
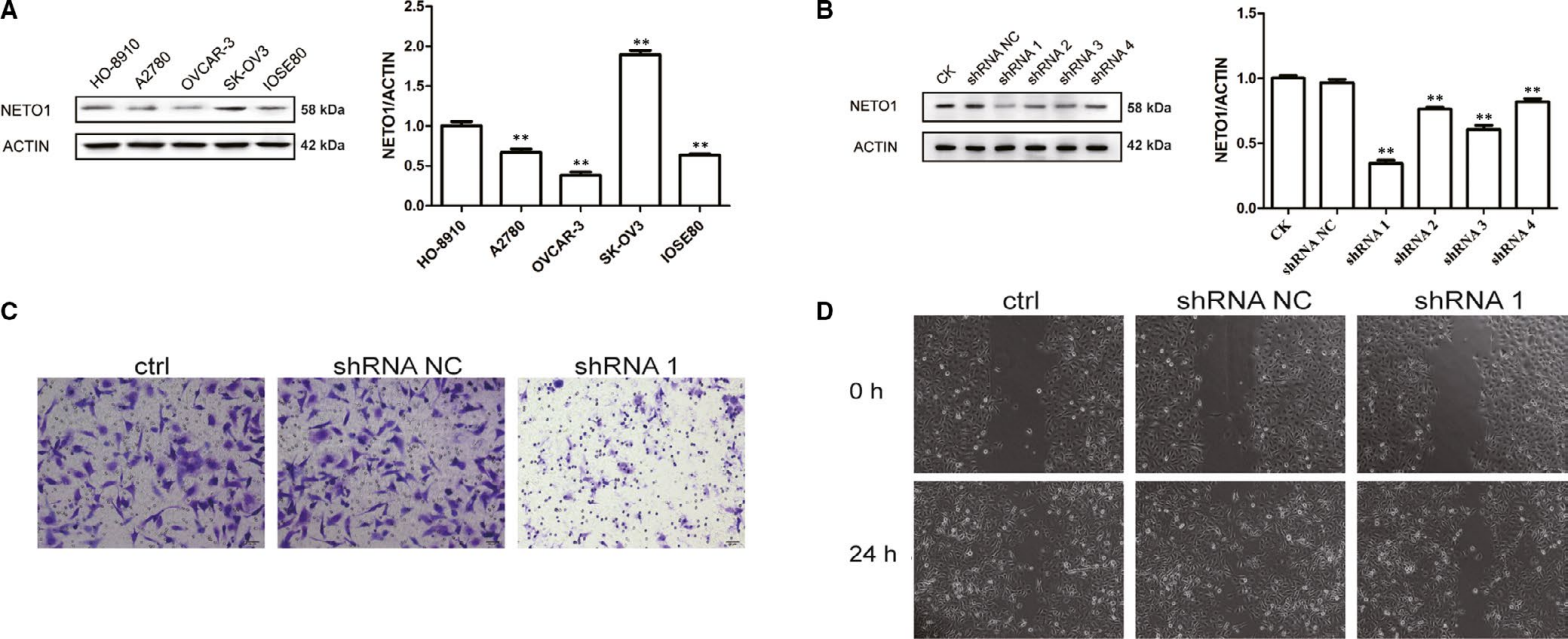
Effect of neuropilin and tolloid‐like 1 (NETO1) on the biological behaviour of ovarian cancer cells. A, The protein expression of NETO1 in five ovarian cell lines. B, The NETO1 protein expression in NETO1‐shRNA‐transfected cells compared with control cells. C, After inhibition of NETO1 expression, there was a decrease in the migration and invasion capability of SK‐OV3 cells. D, After transfecting NETO1‐shRNA1 into SK‐OV3 cells, images were taken at 0 and 24 h

To investigate whether the malignant phenotype of EOC was related to abnormal NETO1 expression, Western blot analysis was conducted to detect the NETO1 protein expression in human EOC cell lines HO‐8910, A2780, SK‐OV3 and OVCAR‐3 and a normal ovarian cell line IOSE80. It was found that NETO1 expression was higher in the four malignant cell lines than in the normal cell line. Western blot results indicated that SK‐OV3 cells had the highest protein expression compared to other cell lines (all *P* < 0.001; Figure 2A). Therefore, the SK‐OV3 cell line with relatively higher NETO1 expression was utilized for cell function experiments.

First, we transfected SK‐OV3 cells with NETO1‐shRNAs and shRNA‐NC. Western blot analysis verified that NETO1 expression levels were reduced drastically in NETO1‐shRNA‐transfected SK‐OV3 cells compared to shRNA‐NC‐transfected cells, with shRNA1 being the most efficient silencer of NETO1 (Figure 2B). Tumour metastasis is known to be related to advanced cancer biology and poor prognosis in patients with EOC. Consequently, we determined whether the overexpression of NETO1 facilitates the metastasis‐related properties of EOC cells in vitro. We used wound healing and transwell assays to evaluate migration and invasion. NETO1 knockdown in SK‐OV3 cells limited the migratory ability in Matrigel‐coated transwell plates and a 200 μL pipette tip created as a wound healing model (Figure 2C,D). These results indicate that NETO1 expression silencing decreased the migration and invasion capability of EOC cells.

### 3.4 Gene set enrichment analysis

**FIGURE 3:**
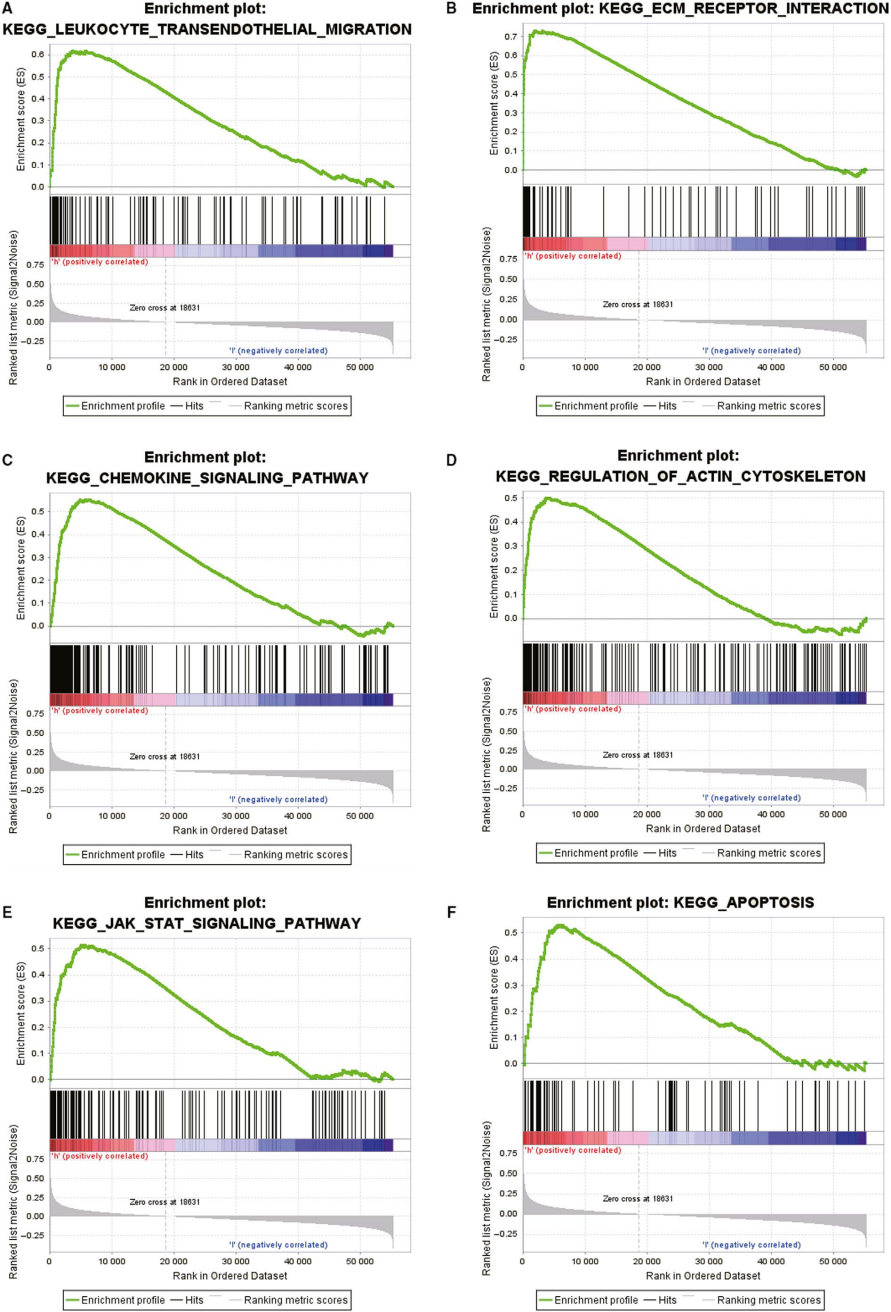
Regulatory mechanisms underlying the functional effects of neuropilin and tolloid‐like 1 (NETO1) using gene set enrichment analysis. A, KEGG LEUKOCYTE TRANSENDOTHELIAL MIGRATION. B, KEGG ECM RECEPTOR INTERACTION. C, KEGG CHEMOKINE SIGNALING PATHWAY. D, KEGG REGULATION OF ACTIN CYTOSKELETON. E, KEGG JAK STAT SIGNALING PATHWAY. F, KEGG APOPTOSIS

To uncover the regulatory mechanisms underlying the functional effects of NETO1 and to identify the signalling pathways differentially activated in EOC, GSEA was performed by comparing high and low NETO1 expression data sets. In the enrichment of MSigDB collection (c2.cp.biocarta and c2.cp.kegg), significant differences were revealed in the GSEA (*P* < 0.05). We selected the most highly enriched signalling pathways according to the NES (Table 5; Figure 3). Figure 3A‐F reveals that leucocyte transendothelial migration, ECM receptor interaction,^13^ chemokine signalling pathway, regulation of actin cytoskeleton,^14^ JAK/STAT signalling pathway^15^ and apoptosis were differentially enriched in the NETO1 high expression phenotype.

**TABLE 5:** Gene sets enrichment

MSigDB collection	Gene set name	NES	FDR
c2.cp.kegg.v6.2.symbols.gmt	KEGG_LEUKOCYTE_TRANSENDOTHELIAL_MIGRATION	2.283	0.000
	KEGG_ECM_RECEPTOR_INTERACTION	2.250	0.000
	KEGG_CHEMOKINE_SIGNALING_PATHWAY	2.035	0.002
	KEGG_REGULATION_OF_ACTIN_CYTOSKELETON	1.960	0.004
	KEGG_JAK_STAT_SIGNALING_PATHWAY	1.942	0.005
	KEGG_APOPTOSIS	1.877	0.009

Note

Gene sets with FDR smaller than 0.05 were considered.

Abbreviations: FDR: false discovery rate; NES, normalized enrichment score.

### 3.5 Regulation of actin cytoskeleton

**FIGURE 4:**
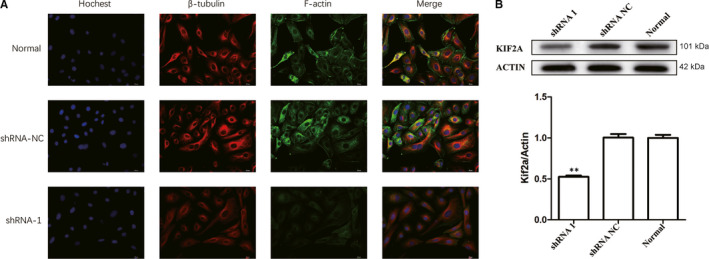
Regulation of actin cytoskeleton, a possible signalling pathway that might be involved in development of epithelial ovarian cancer. A, Immunofluorescence microscopy shows that SK‐OV3 cells expressing neuropilin and tolloid‐like 1 (NETO1)‐shRNA1 have reduced levels of β‐tubulin and F‐actin expression. B, Western blot analysis demonstrates that SK‐OV3 cells expressing NETO1‐shRNA1 have reduced KIF2A protein level

On the basis of evidence from GSEA, we chose regulation of actin cytoskeleton as a possible signalling pathway that might be involved in development of EOC. Immunofluorescence microscopy indicated that cells expressing NETO1‐shRNA had reduced levels of β‐tubulin and F‐actin expression, a key signalling protein in the regulation of actin cytoskeleton (Figure 4A). Furthermore, Western blot analysis revealed that cells expressing NETO1‐shRNA exhibited reduced KIF2A expression levels, which is a motor protein engaged in transporting cargo proteins along the microtubules (MTs; Figure 4B), suggesting that NETO1 is an upstream regulatory element for KIF2A and affects tumour biology through the regulation of actin cytoskeleton in EOC.

## 4 DISCUSSION

Epithelial ovarian cancer is the most lethal malignancy of the female reproductive system. Only about 30% of EOC patients have tumours confined to the ovary, and majority of the patients are diagnosed in the advanced stage when the tumour has spread to the pelvic and abdominal organs. Consequently, even after the required surgery depending on the condition of the EOC patient and post‐surgical chemotherapy, the 5‐year survival rate remains between 20% and 30%.^16^ Recent research has reported that 21 genes inclusive of NETO1 are associated with bowel metastases in EOC. Moreover, NETO2, an important paralog of NETO1, has been investigated as a tumour‐promoting molecule in a variety of cancers and NETO2 expression has been found to have a significant correlation with poor prognostic results in these cancers. In studies on HCC, it was reported that neoangiogenesis‐related genes including NETO2 and four other hepatic gene signatures may predict fast‐growing HCC and poor survival.^6^ In case of prostate cancers, NETO2 expression has been found to be closely associated with tumour heterogeneity which is related to therapeutic response, chemotherapy resistance and the consequent risk of poor outcome. Previous studies have demonstrated that NETO2 promoted gastric cancer cell invasion and metastasis via activation of the PI3K/Akt/NF‐κB/Snail axis.^17^ Furthermore, aberrant expression of NETO2 has been observed in other types of carcinomas including pancreatic cancer, glioma, nasopharyngeal carcinoma, renal clear cell carcinoma, colorectal cancer and lung adenocarcinoma and represents not only a novel prognostic indicator but also a potential therapeutic target. However, to date, little information is available about the potential impact of NETO1 on metastasis and survival in EOC patients. Therefore, this is the first study to illustrate the potential role of NETO1 in EOC.

First, we observed the expression of NETO1 in human ovary benign and malignant tissue samples. NETO1 protein overexpression, observed in cases of EOC, may serve as an independent predictive factor for poor outcome in patients with EOC. Subsequently, experiments on a panel of EOC cell lines indicated that the silencing of NETO1 expression decreased the metastatic ability of cells which has a relation with advanced cancer biology. We further aimed to explore the potential mechanism and functional effects of NETO1 on downstream molecules involved in carcinogenesis. Bioinformatic analysis was utilized to identify the signalling pathways differentially activated in EOC involving different levels of NETO1. On the basis of evidence from GSEA, we chose regulation of actin cytoskeleton as a possible signalling pathway that might be involved in development of EOC. In particular, regulation of actin cytoskeleton dynamics may also be involved in the progression of cell division, motility and polarization, which might be related to aggressive tumour behaviour. β‐tubulin and actin filaments, which are major components of the cytoskeleton in eukaryotic cells, play essential roles in tumour cell invasion and metastasis. RNA‐mediated knockdown of NETO1 reduced the expression of actin cytoskeleton regulation‐related proteins (β‐tubulin, F‐actin) and mitotic centromere‐associated kinesin‐13 protein KIF2A.

Our recent study established that KIF2A, a protein suppressed by miR‐206, is relevant in the poor prognosis of ovarian cancer.^18^ Notably, KIF2A is dysregulated in several other types of human cancers and functions as an oncogene in oral squamous cell carcinoma,^19^ breast cancer,^20^ human glioma,^21^ gastric cancer^22^, ^23^ and lung adenocarcinoma.^24^ In our study we discovered that NETO1 silencing, causing inactivation of actin cytoskeleton, decreased the expression of Kinesin‐13 protein KIF2A, suggesting an interaction between NETO1 and KIF2A in EOC. The KIFs have a critical role in spindle orientation and chromosomal movements during the process of mitosis and cytoskeleton reorganization.^25^, ^26^, ^27^ Three distinct genes, including KIF2A, KIF2B and MCAK/ KIF2C, encode the kinesin‐13 family members in the human genome, that participate in the processes of intracellular transport, bipolar spindle assembly and cell division.^28^, ^29^, ^30^ KIF2A specifically localizes to centrosomes during mitosis and encodes a motor protein which is engaged in transporting cargo proteins along MTs. It is known conclusively that MTs play a vital part in cellular events involved in cell migration.^31^ KIF2A has been identified to be a MT‐depolymerizing kinesin. The findings of our study suggest that NETO1 might to be a novel protein that interacts with KIF2A. Furthermore, NETO1 depletion inhibits the ability of KIF2A to promote tumour progression. Aberrant expression of NETO1 leads to activation of the KIF2A protein, and overactivation of KIF2A protein might result in an imbalance of the protein‐protein interactions. This might promote depolymerization of MT that is associated with KIF2A and ultimately cause infinite transmission of signals. NETO1 depletion reduces the expression of cytoskeleton‐related genes and impairs SK‐OV3 cell invasion and migration. These results suggest that NETO1 induces cytoskeletal reorganization that subsequently promotes tumour cell invasion and metastasis.

To sum up, our study revealed that NETO1 might enhance migration and invasive potential of EOC, modulate the expression of cytoskeletal genes and promote tumour development, progression and metastasis. Therefore, NETO1 may serve as a potential biomarker to identify a subgroup of patients with a more aggressive phenotype of ovarian cancer. However, our study has some limitations such as it only involved vitro experiments. Consequently, there is an urgent need to perform further investigations to verify whether NETO1 modulates EOC behaviour in vivo experiments performed on an EOC animal model and to further evaluate the clinical applications of the anti‐actin cytoskeleton mechanisms in EOC.

## CONFLICT OF INTEREST

The authors confirm that there are no conflicts of interest.

## AUTHOR CONTRIBUTIONS

**Yunzhao Xu:** Conceptualization (lead); Data curation (lead); Writing‐original draft (lead). **Wei Wang:** Conceptualization (supporting); Writing‐original draft (supporting); Writing‐review & editing (equal). **Jinling Chen:** Supervision (supporting). **Haixia Mao:** Data curation (supporting); Formal analysis (equal). **Yuanlin Liu:** Methodology (lead). **Shuting Gu:** Methodology (supporting). **Qinqin Liu:** Methodology (supporting). **Qinghua Xi:** Supervision (lead). **Wenyu Shi:** Funding acquisition (equal); Resources (lead).

## Data Availability

The data that support the findings of this study are available from the corresponding author upon reasonable request. The public data that support the findings of this study are openly available in GEO database at (https://www.ncbi.nlm.nih.gov/).^12^
